# Supplementary material for: Systematic HOIP interactome profiling reveals critical roles of linear ubiquitination in tissue homeostasis
Source: Nat Commun. 2024 Apr 6;15:2974. doi: 10.1038/s41467-024-47289-2 (PMC10998861; doi:10.1038/s41467-024-47289-2)
Supplement: Supplementary file 1 — Supplementary Information [file 41467_2024_47289_MOESM1_ESM.pdf]

Supplementary Information File

Systematic HOIP Interactome Profiling Reveals Critical Roles of Linear Ubiquitination in Tissue Homeostasis

Yesheng Fu, Lei Li, Xin Zhang, Zhikang Deng, Ying Wu, Wenzhe Chen, Yuchen Liu, Shan He, Jian Wang, Yuping Xie, Zhiwei Tu, Yadi Lyu, Yange Wei, Shujie Wang, Chun-Ping Cui, Cui Hua Liu*, Lingqiang Zhang*

* Correspondence: zhanglq@nic.bmi.ac.cn (L.Z.) & liucuihua@im.ac.cn (C.H.L.)

The PDF file includes:
Supplementary Figures 1–9
Unprocessed blots for Supplementary Figures

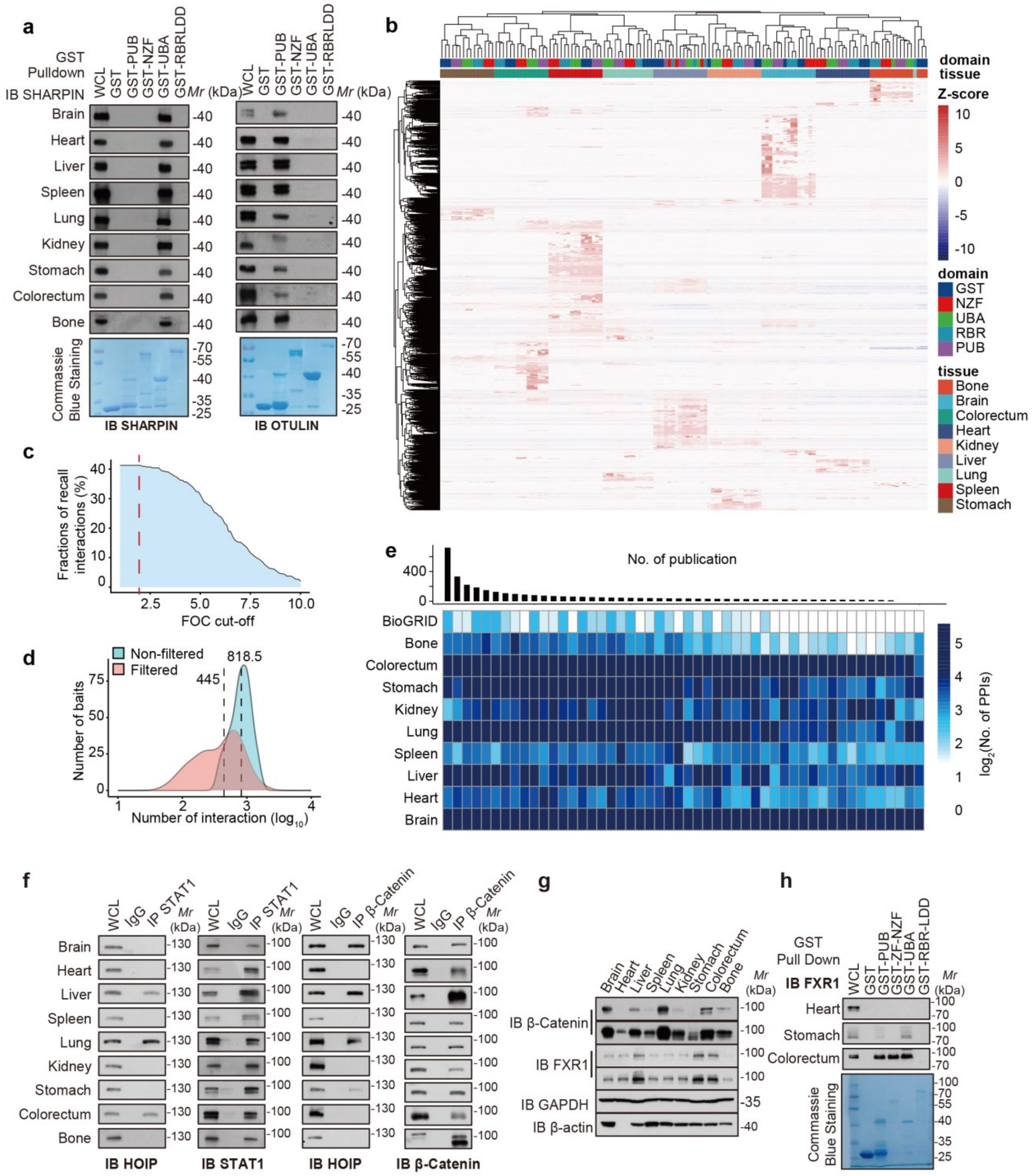

Supplemental Fig. 1 Data filtering and detection of known interactions, related to Fig. 1.

a Immunoprecipitation analysis of canonical interactions, such as OTULIN and SHARPIN between four HOIP domains in various tissues.

b Global visualization of HOIP interacting proteins across domains and tissues. Protein abundance is calculated by iBAQ and homogenized by z-score.

c Positive discovery rate of the known HOIP interacting proteins with different fold of change (FOC)

cut-offs.

d Histograms depicting the number of HOIP interactions before and after filtering.

e Number of interactions between proteins binned by number of publications. Histogram shows the median number of publications.

f Endogenous immunoprecipitation analysis of STAT1 (or β-Catenin) and HOIP across tissues.

g Immunoblot analysis of HOIP, β-Catenin and FXR1 across tissues.

h Immunoprecipitation analysis of the known β-Catenin-interacting protein FXR1 between four HOIP domains in heart, stomach and colorectum.

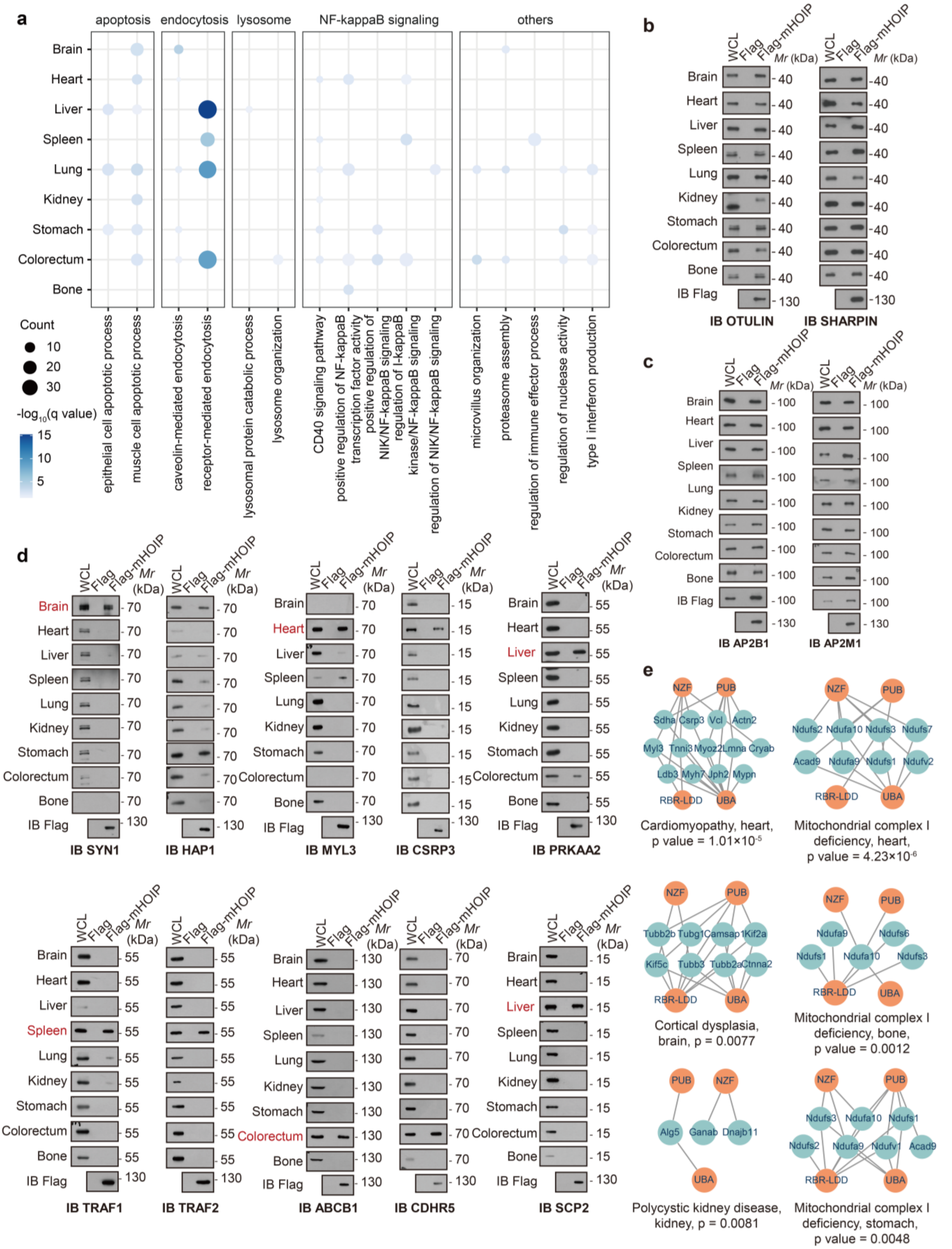

Supplemental Fig. 2 Identification of tissue-specific HOIP PPIs, related to Fig. 2.

a Functional enrichment analysis of HOIP PPIs identifies a number of Gene Ontology (GO) terms

associated with the known functions of HOIP. q value is calculated by Benjamini–Hochberg method.

b Immunoprecipitation of ectopic expressed HOIP from HEK293T cells in different tissues and immunoblot with the antibodies of OTULIN and SHARPIN. The ectopically expressed Flag-tagged only peptide was a negative control.

c Immunoprecipitation of ectopic expressed HOIP from HEK293T cells in different tissues and immunoblot with the antibodies of AP2B1 and AP2M1. The ectopically expressed Flag-tagged only peptide was a negative control.

d Immunoprecipitation of ectopic expressed HOIP from HEK293T cells in different tissues and immunoblot with the antibodies of tissue-specific proteins. The ectopically expressed Flag-tagged only peptide was a negative control.

e HOIP interaction modules, in which interactors with the same disease term are present at a statistically significant frequency, are shown. p values are from the Fisher exact test.

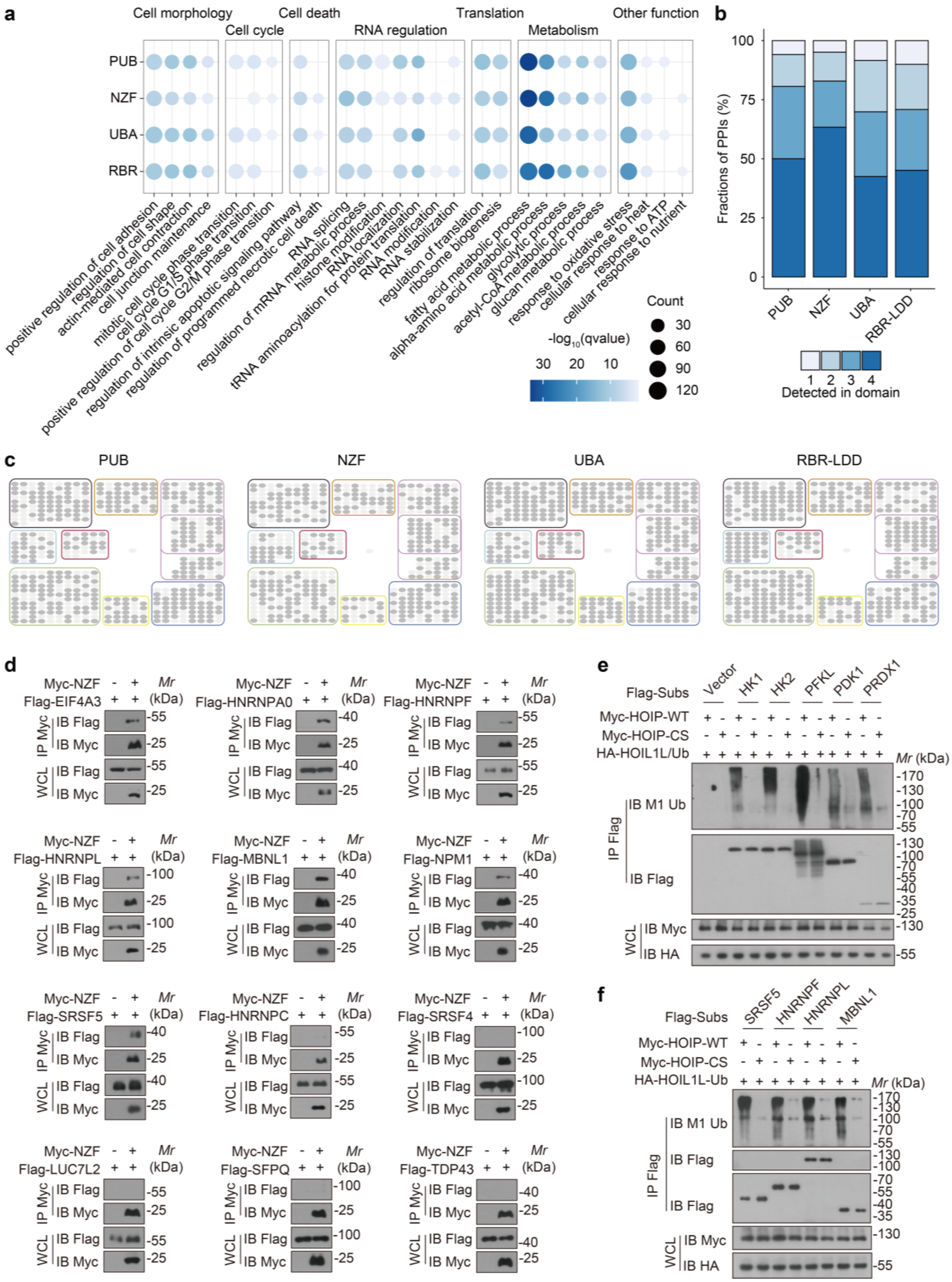

Supplemental Fig. 3 Functional landscape of proteins included in the HOIP interaction network,

related to Fig. 3.

a Functional enrichment analysis of HOIP PPIs identifies a number of Gene Ontology (GO) terms associated with the fundamental cellular functions. q value is calculated by Benjamini–Hochberg method.

b Distribution of the fraction of HOIP PPIs quantified in each domain.

c Individual diagrams of domain-dependent interactors. Domain-dependent nodes are shown in gray, whereas domain-independent interactors are depicted in white.

d Immunoprecipitation of ectopic expressed Myc-tagged HOIP NZF in lysates from HEK293T cells transfected with indicate Flag-tagged proteins, and immunoblot with the corresponding antibodies. The Myc-tagged vector was a negative control.

e Immunoprecipitates of HK1, HK2, PFKL, PDK1 and PRDX1 to detect the linear ubiquitination in HEK293T cells transfected with Myc-tagged HOIP WT or CS (a HOIP inactivate mutant).

f Immunoprecipitates of SRSF5, HNRNPF, HNRNPL and MBNL1 to detect the linear ubiquitination in HEK293T cells transfected with Myc-tagged HOIP WT or CS (a HOIP inactivate mutant).

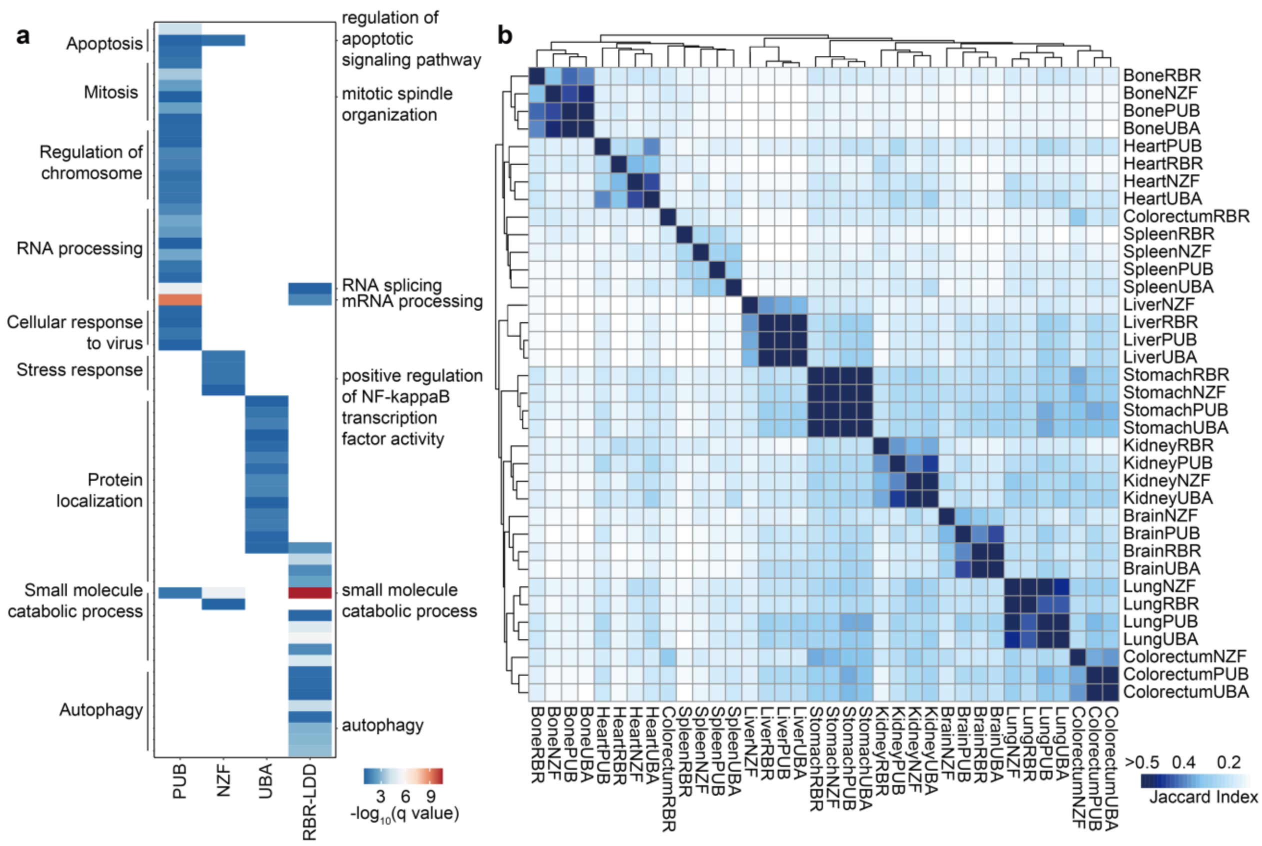

Supplemental Fig. 4 Functional enrichment of domain-specific HOIP PPIs across tissues, related to Fig. 3.

a Functional enrichment analysis of HOIP PPIs identifies a number of Gene Ontology (GO) terms associated with the domain-specific PPIs. q value is calculated by Benjamini–Hochberg method.

b Jaccard index of HOIP PPIs detected by various tissues and domains to show protein coverage between different tissues and domains.

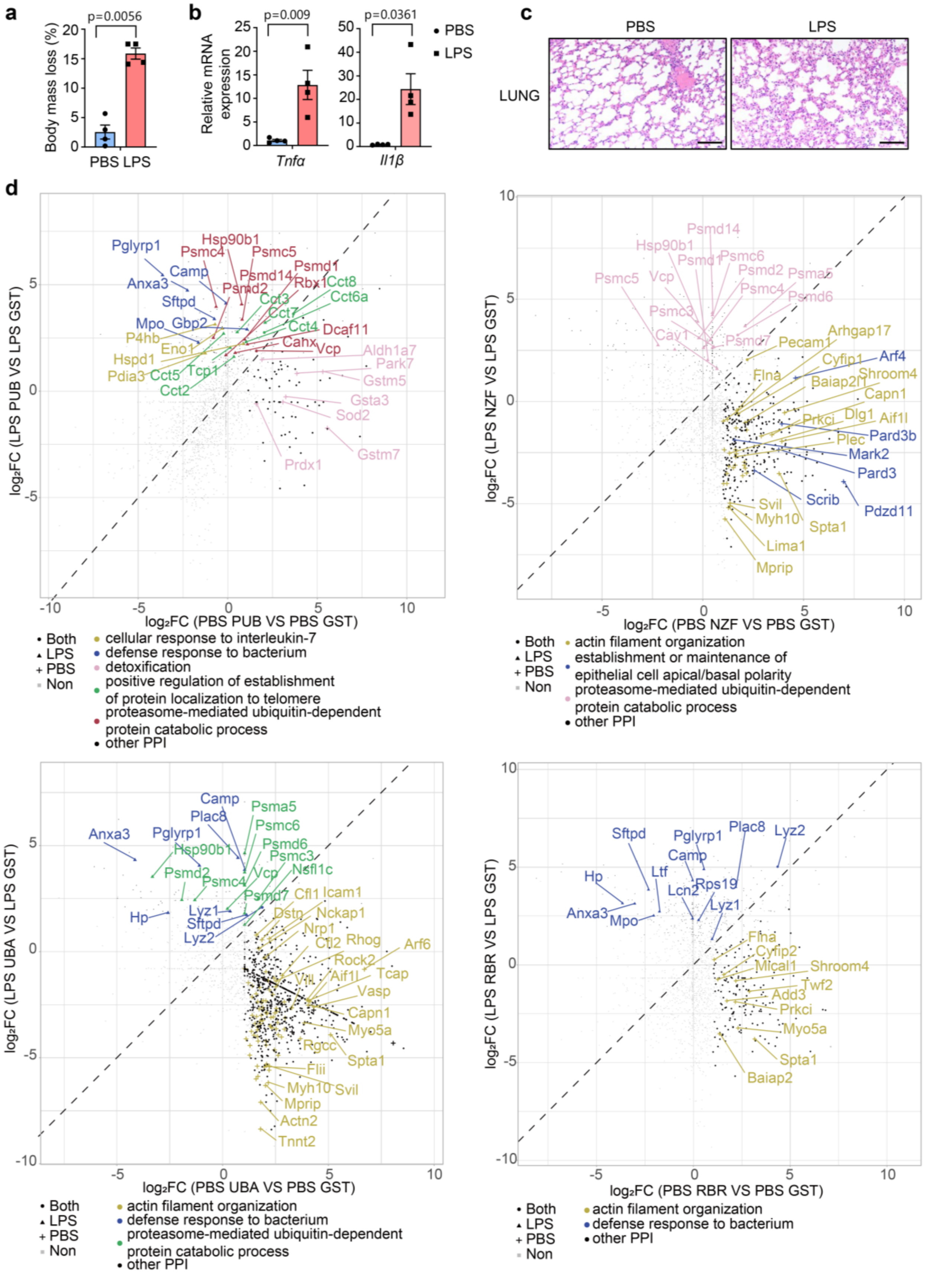

Supplemental Fig. 5 Functional landscape of HOIP interaction network in lungs from LPS-induced sepsis models, related to Fig. 3.

a Weight monitoring of mice i.p. injection with or without LPS (12 mg/kg body weight in saline). n = 4 per group.

b Quantitative RT-PCR analysis of *Tnfα* and *IL1β* mRNA levels in lungs from LPS-induced sepsis models. n = 4 per group.

c H&E staining of lungs from LPS-induced sepsis models. Scale bars, 100 μm.

d Correlation plot of $\log_2$FC (HOIP domains/GST) for lungs with or without LPS injection.

Data are shown as the mean ± SEM; p values are from the unpaired two-sided t-test.

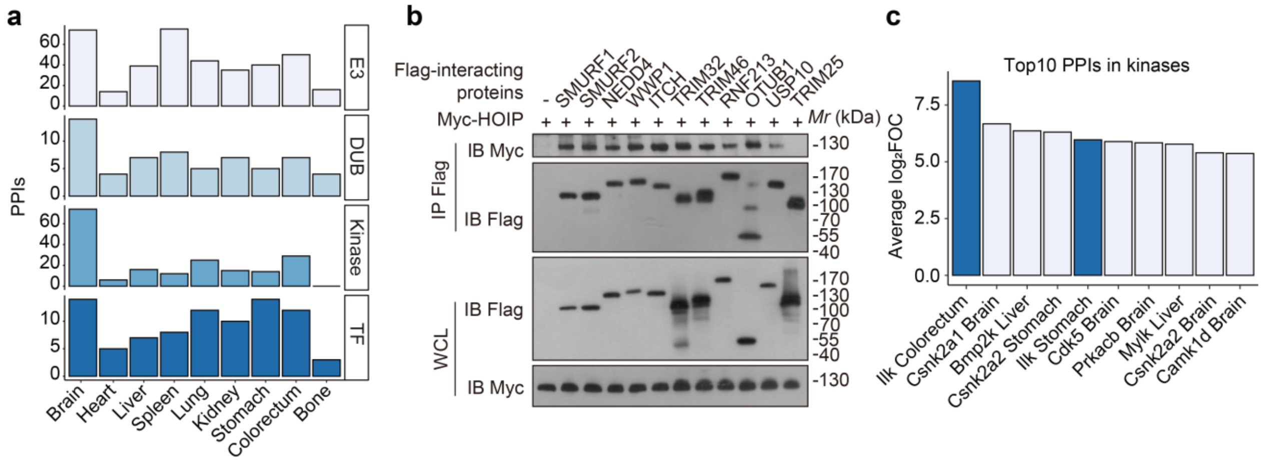

Supplemental Fig. 6 Functional analysis of HOIP PPIs associated with kinases, E3 ligases, deubiquitinases and transcriptional factors, related to Fig. 4.

a Barchart with the numbers of HOIP PPIs associated with kinases, E3 ligases, deubiquitinases and transcriptional factors.

b Immunoprecipitation of ectopic expressed NEDD4-1, WWP1, ITCH, SMURF1, SMURF2, TRIM32, TRIM46, RNF213, OTUB1 and USP10 in HEK293T cells transfected with Myc-tagged HOIP and immunoblot with indicated antibodies.

c Representable top 10 HOIP PPIs in the kinases.

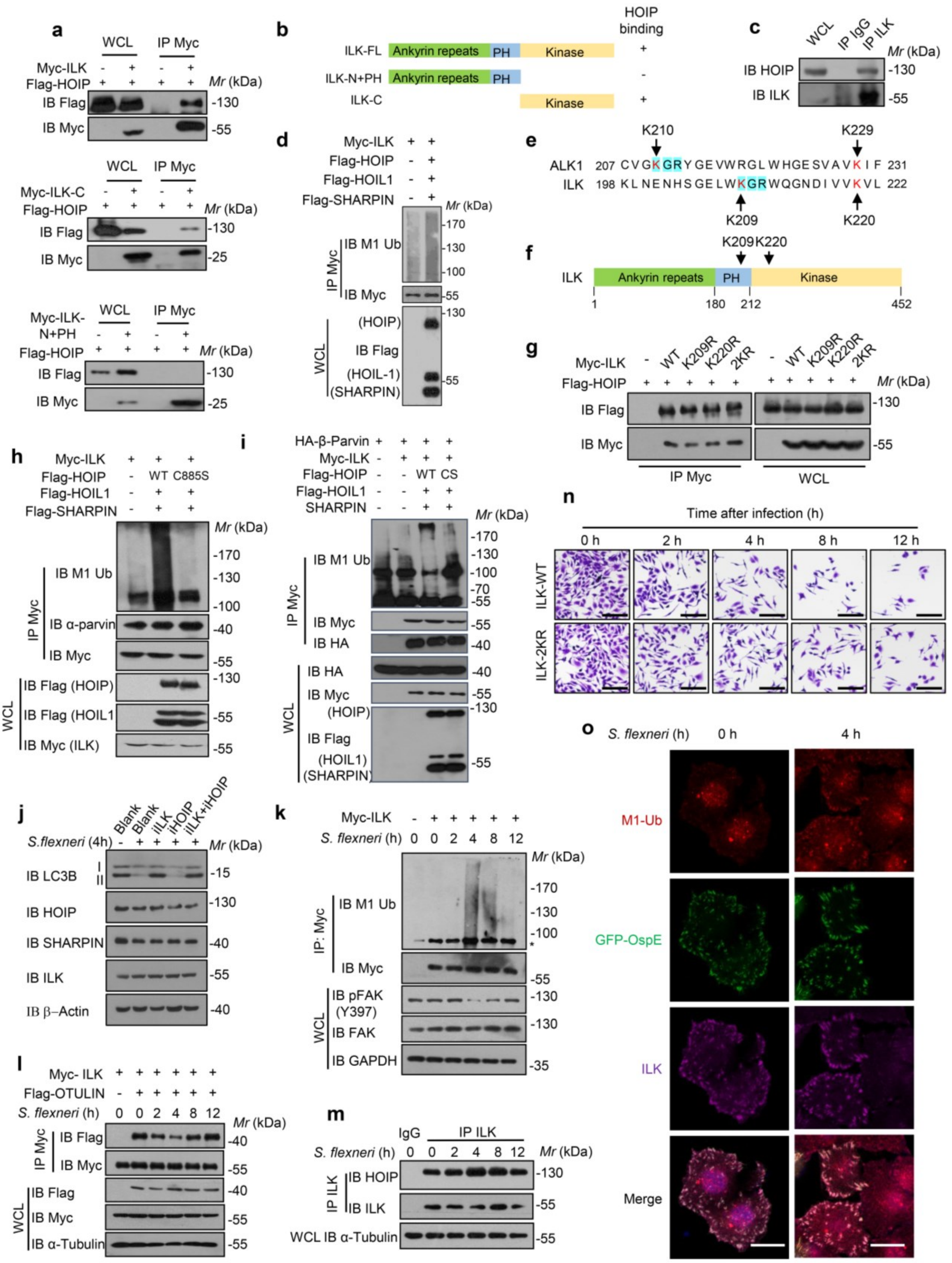

Supplemental Fig. 7 Linearly ubiquitinated ILK is dynamically regulated during *Shigella flexner* infection, related to Fig. 4.

a HEK293T cells were transfected with Flag-tagged HOIP and Myc-tagged ILK (or ILK truncations), and then immunoprecipitated with the Myc antibody, followed by immunoblotting with the indicated

antibodies.

b Schematic representative of the ILK truncations.

c Immunoprecipitation of ILK in HEK293T cells and immunoblot with indicated antibodies.

d Immunoprecipitation of ILK linear ubiquitination in HEK293T cells transfected with Flag-tagged HOIP, Flag-tagged HOIL1, Flag-tagged SHARPIN and immunoblot with indicated antibodies.

e Distribution diagram of potential linear ubiquitination sites of ILK.

f Sequence alignment of ILK and ALK1. The horizontal line represents a conservative sequence, arrows indicate linear ubiquitination sites of ALK1 and predicted potential linear ubiquitination sites of ILK.

g Immunoprecipitation of ILK and its mutants in HEK293T cells transfected with Myc-tagged ILK wild type (WT) or its mutants K209R, K220R or 2KR (K209R/K220R) and Flag-tagged HOIP, followed by immunoblotting with indicated antibodies.

h Cell lysates from HEK293T cells transfected with Myc-tagged ILK, Flag-tagged HOIL-1, Flag-tagged SHARPIN and Flag-tagged HOIP wild type or its catalytically inactivated mutant were immunoprecipitated with the Myc antibody, followed by immunoblotting with indicated antibodies.

i Cell lysates from HEK293T cells transfected with HA-tagged β-Parvin, Myc-tagged ILK, Flag-tagged HOIL-1, Flag-tagged SHARPIN and Flag-tagged HOIP wild type or its catalytically inactivated mutant were immunoprecipitated with the Myc antibody, followed by immunoblotting with indicated antibodies.

j Immunoblot analysis of the LC3B in *Shigella flexner* infected Hela cells with 2 μM ILK inhibitor ILK-IN-3 and 20 μM HOIP inhibitor HOIPin-8.

k Immunoprecipitation of ILK linear ubiquitination in Myc-tagged ILK overexpressed HeLa cells, infected with *Shigella flexner* for 0 h, 2 h, 4 h, 8 h and 12 h respectively. And immunoblot with indicated antibodies.

l Immunoprecipitation of ILK in HeLa cells transfected with Flag-tagged OTULIN and Myc-tagged ILK after *Shigella flexner* infection and immunoblot with indicated antibodies.

m Immunoprecipitation analysis of interactions between ILK and HOIP after *Shigella flexner* infection in HeLa cells.

n Representative images showing the attached cells of control and ILK-2KR groups infected with *Shigella flexner* for indicated time points, Scale bars, 10 μm.

o HeLa cells were infected with or without *Shigella flexner* for 4h, then the cells were collected for immunofluorescence with indicated antibodies. Scale bars, 20 μm.

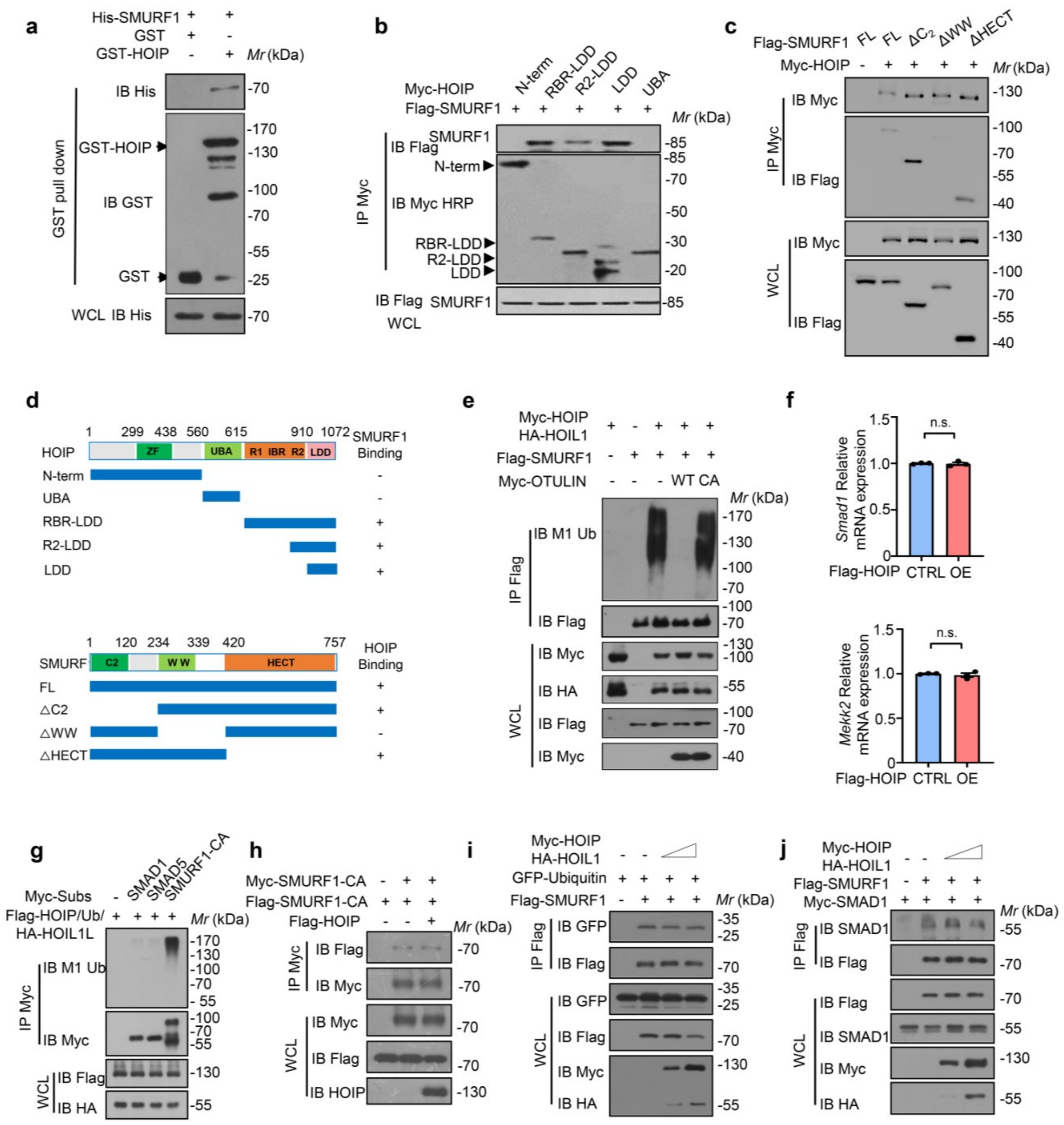

Supplemental Fig. 8 SMURF1 is a HOIP-interacting protein, related to Fig. 5.

a In *vitro* binding assay of purified GST-tagged HOIP and His-tagged SMURF1.

b Immunoprecipitates of HOIP and its truncations in HEK293T cells transfected with Flag-tagged SMURF1 and immunoblot with the indicated antibodies.

c Immunoprecipitates of SMURF1 and its truncations in HEK293T cells transfected with Myc-tagged HOIP and immunoblot with the indicated antibodies.

d Schematic representative of the HOIP-interacting region in SMURF1 and the SMURF1-interacting region in HOIP.

e Immunoprecipitates of SMURF1 linear ubiquitination in HEK293T cells transfected with LUBAC and OTULIN mutants.

f Analysis of *Smad1* and *Mekk2* genes expression by qPCR in HOIP stably over-expressed (OE) BMSCs.

g Immunoprecipitates of SMAD1, SMAD5 and SMURF1-C699A's linear ubiquitination in HEK293T cells transfected with LUBAC

h Immunoblot analysis of the interaction between Myc-tagged SMURF1-C699A and Flag-tagged SMURF1-C699A in HEK293T cells transfected with Myc-tagged HOIP.

i Immunoblot analysis of the interaction between SMURF1 and ubiquitin in HEK293T cells transfected with LUBAC.

j Immunoblot analysis of the interaction between SMURF1 and its substrate SMAD1 in HEK293T cells transfected with LUBAC.

Data are shown as the mean ± SEM; n = 3 per group, p values are from the unpaired two-sided t-test. n.s., no significant.

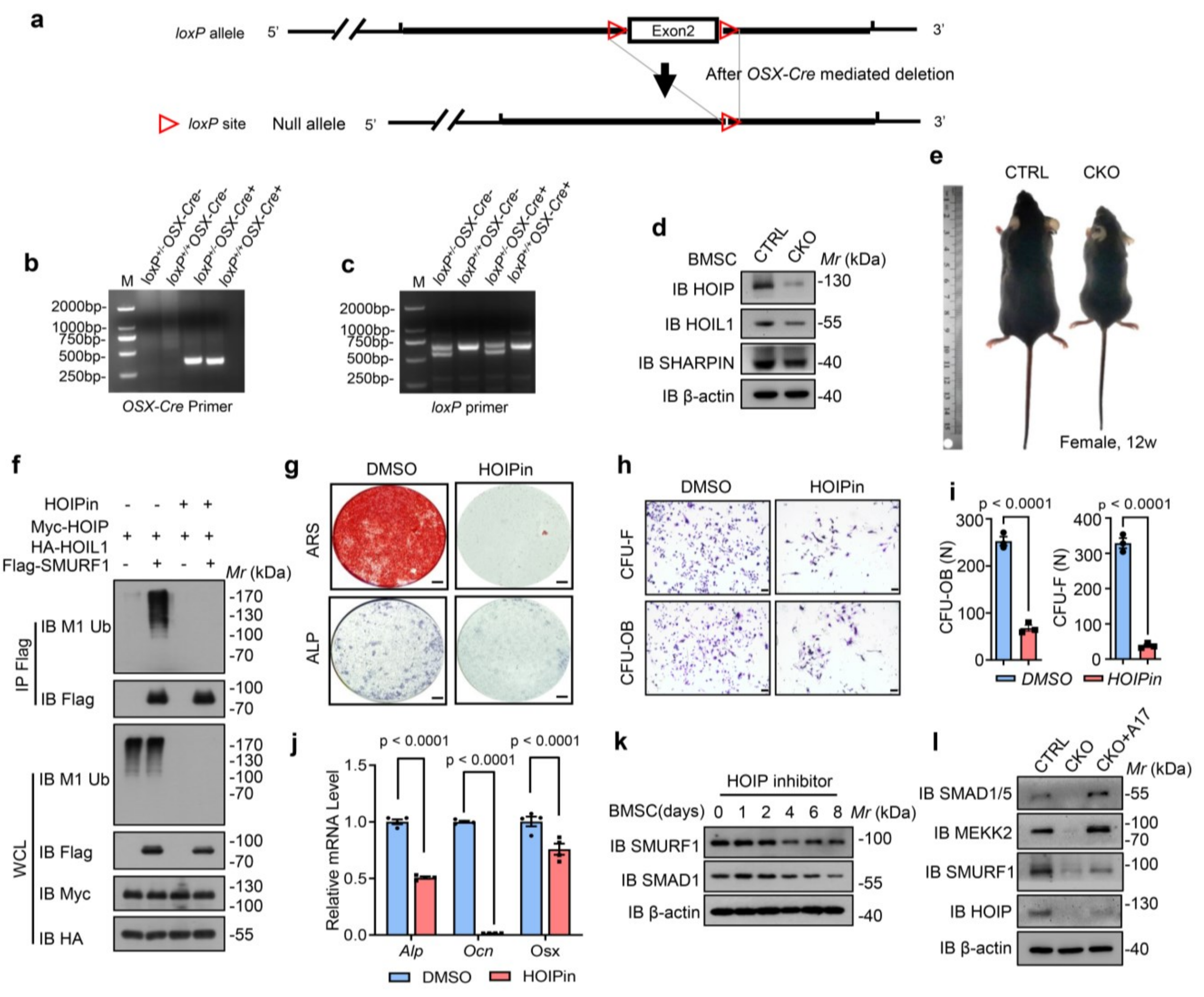

Supplemental Fig. 9 Targeted disruption of the murine *Hoip* gene, related to Fig. 6.

a Diagram of *Hoip* osteoblast specific knockout strategy.

b, **c** PCR analysis of HOIP CTRL and CKO mice tails.

d Immunoblot analysis of HOIP, HOIL1 and SHARPIN protein levels in BMSCs from HOIP CTRL and CKO mice.

e Representative image of 8-week-old female HOIP CTRL and CKO mice.

f Immunoprecipitates of SMURF1 linear ubiquitination in HEK293T cells transfected with LUBAC with or without HOIP inhibitor HOIPin-8.

g Representative images of ALP and ARS staining of BMSCs with or without HOIP inhibitor treatment after cultured in osteogenic medium for 14 and 28 days. Scale bars, 2 mm.

h, i Representative images and quantification of CFU-OB and CFU-F of BMSCs with or without HOIP inhibitor treatment. Scale bars, 50 µm. n = 3 per group.

j Immunoblot of SMURF1 in BMSCs with HOIP inhibitor treatment.

k Quantitative RT-PCR analysis of osteogenesis genes mRNA levels in BMSCs with or without HOIP inhibitor treatment. n = 3 per group.

l Immunoblot of SMURF1 and its substrate SMAD1/5, MEKK2 in BMSCs from CTRL, CKO and CKO treated with SMURF1 inhibitor A17 groups for 28 days.

Data are shown as the mean ± SEM; p values are from the unpaired two-sided t-test and the two-way ANOVA (Sidak's multiple comparisons test).

Uncropped blots for Supplementary Figures

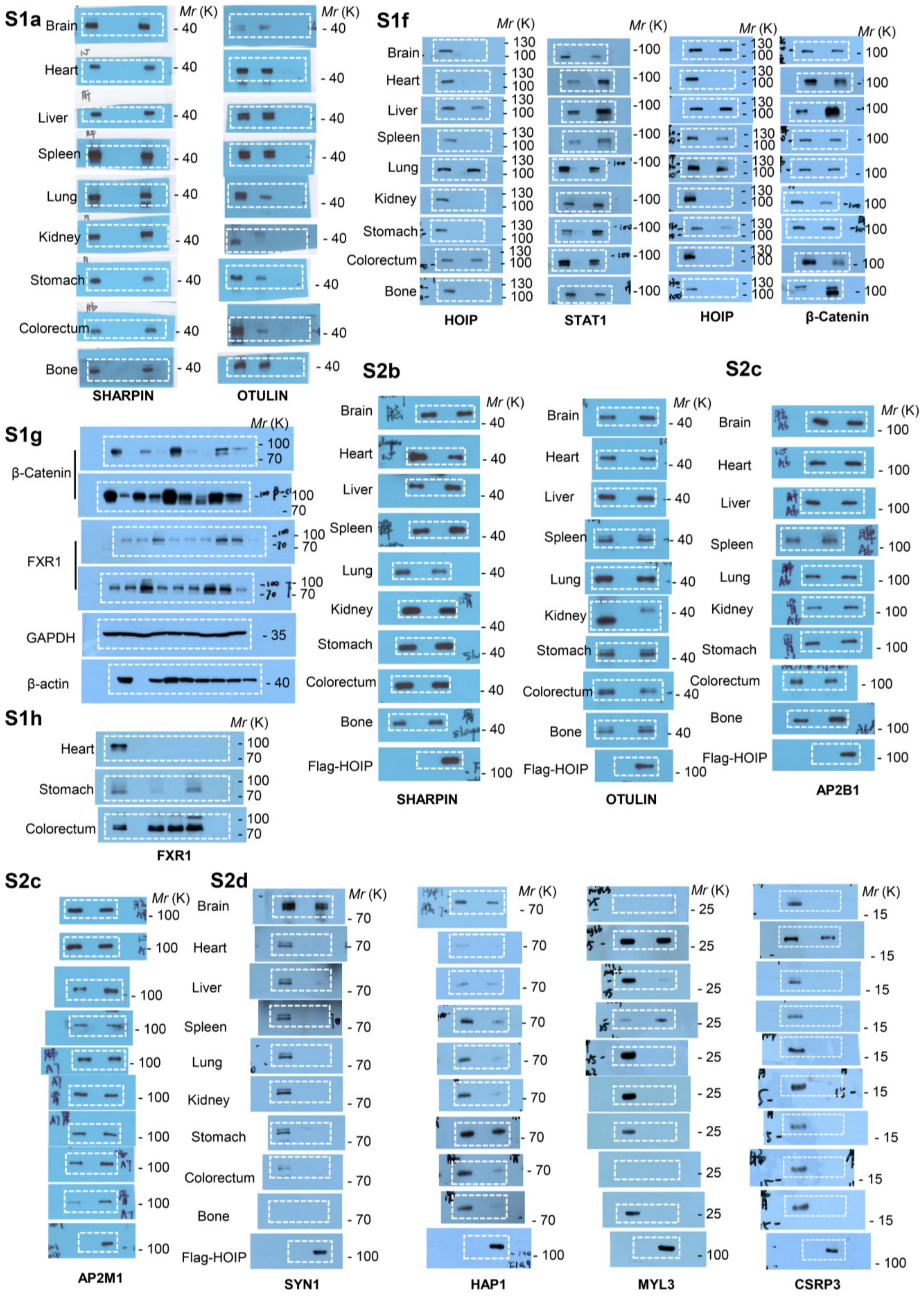

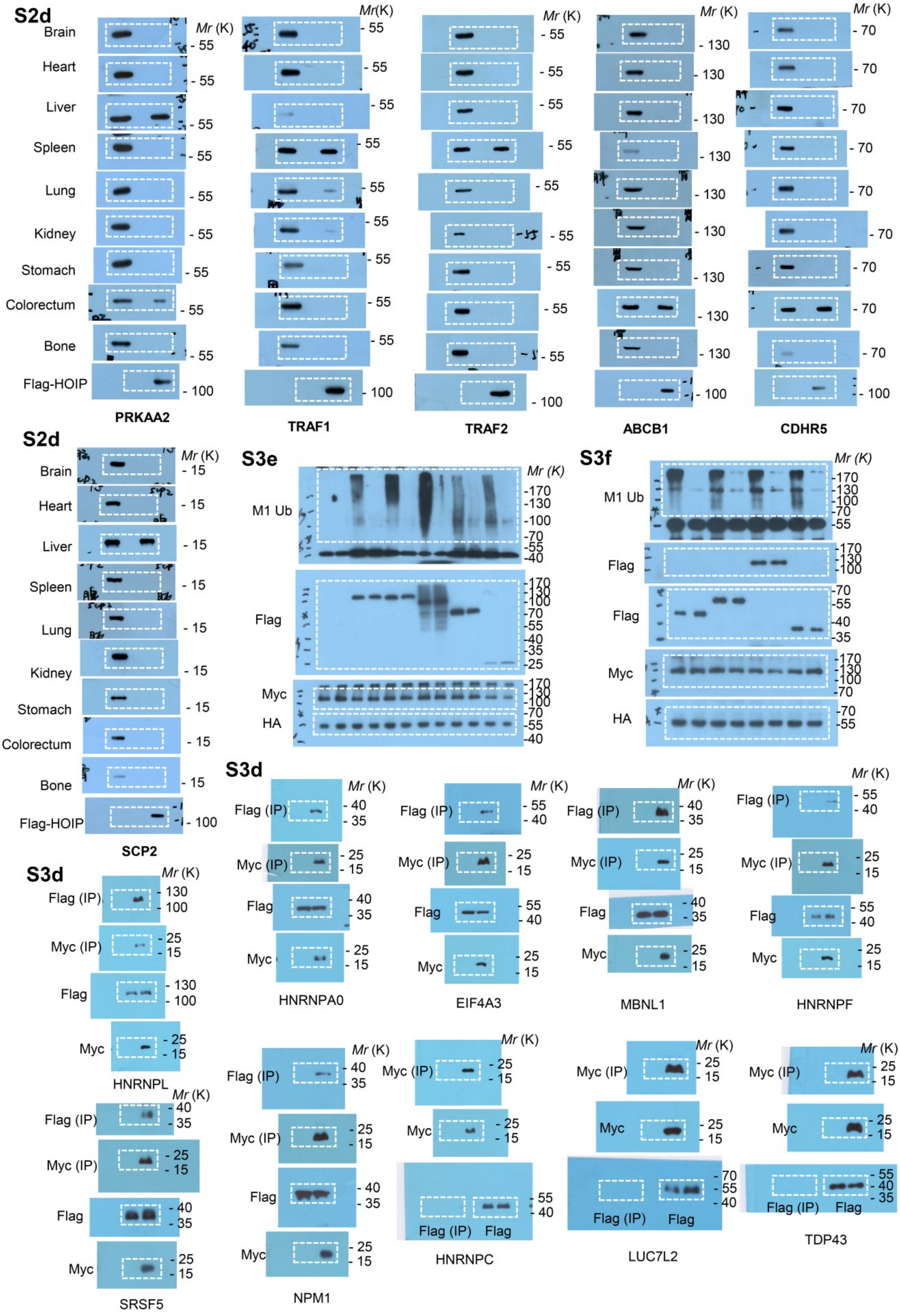

S2d
Brain
Heart
Liver
Spleen
Lung
Kidney
Stomach
Colorectum
Bone
Flag-HOIP
Mr (K)
- 55
- 100
- 130
- 70
PRKAA2
TRAF1
TRAF2
ABCB1
CDHR5
S2d
- 15
SCP2
S3e
M1 Ub
Flag
Myc
HA
-170
-130
-100
-70
-55
-40
-35
-25
S3f
M1 Ub
Flag
Flag
Myc
HA
S3d
Flag (IP)
Myc (IP)
Flag
Myc
HNRNPL
SRSF5
HNRNPA0
NPM1
EIF4A3
HNRNPC
MBNL1
LUC7L2
HNRNPF
TDP43
Flag (IP)
Flag
- 40
- 35
- 25
- 15
- 130
- 100
- 55

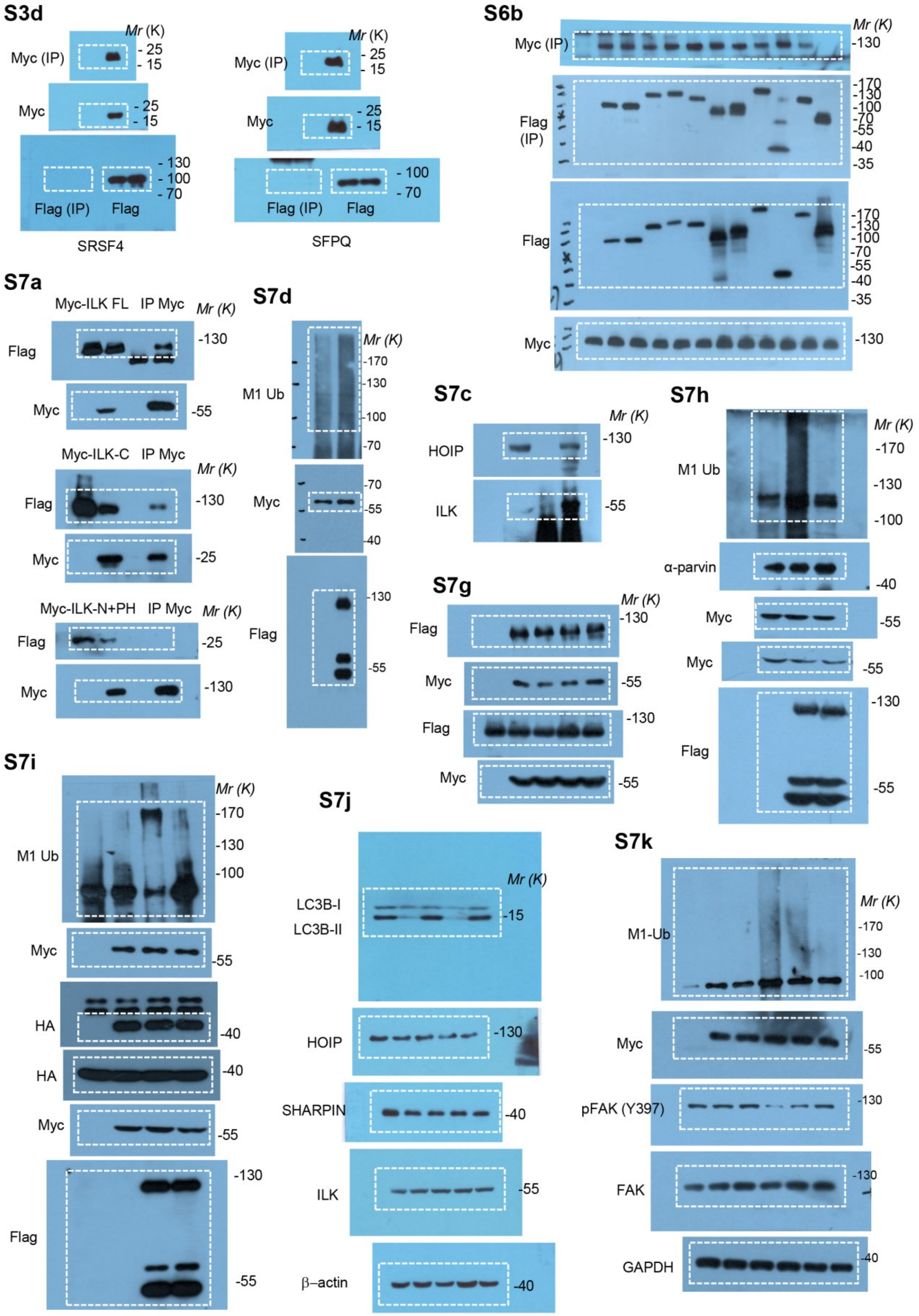
S3d
Mr (K)
Myc (IP)
- 25
- 15
Myc
- 25
- 15
- 130
- 100
- 70
Flag (IP)
Flag
SRSF4
Mr (K)
Myc (IP)
- 25
- 15
Myc
- 25
- 15
- 100
- 70
Flag (IP)
Flag
SFPQ
S6b
Mr (K)
Myc (IP)
-130
Flag (IP)
-170
-130
-100
-70
-55
-40
-35
Flag
-170
-130
-100
-70
-55
-40
-35
Myc
-130
S7a
Myc-ILK FL
IP Myc
Mr (K)
Flag
-130
Myc
-55
Myc-ILK-C
IP Myc
Mr (K)
Flag
-130
Myc
-25
Myc-ILK-N+PH
IP Myc
Mr (K)
Flag
-25
Myc
-130
S7d
Mr (K)
M1 Ub
-170
-130
-100
-70
Myc
-70
-55
-40
Flag
-130
-55
S7c
Mr (K)
HOIP
-130
ILK
-55
S7h
Mr (K)
M1 Ub
-170
-130
-100
α-parvin
-40
Myc
-55
Myc
-55
Flag
-130
-55
S7g
Mr (K)
Flag
-130
Myc
-55
Flag
-130
Myc
-55
S7i
Mr (K)
M1 Ub
-170
-130
-100
Myc
-55
HA
-40
HA
-40
Myc
-55
Flag
-130
-55
S7j
Mr (K)
LC3B-I
LC3B-II
-15
HOIP
-130
SHARPIN
-40
ILK
-55
β–actin
-40
S7k
Mr (K)
M1-Ub
-170
-130
-100
Myc
-55
pFAK (Y397)
-130
FAK
-130
GAPDH
-40

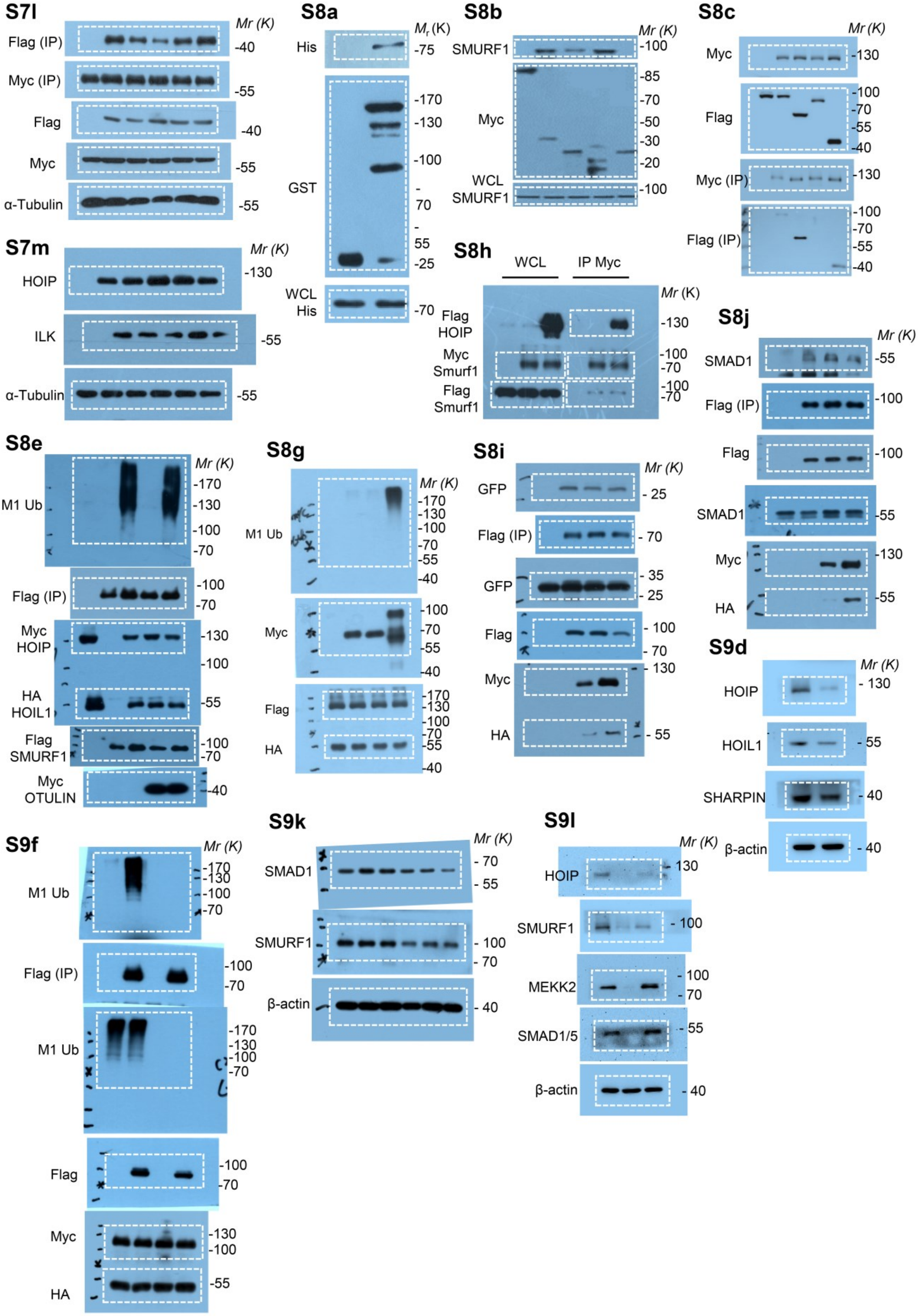

S7l
Mr (K)
Flag (IP)
-40
Myc (IP)
-55
Flag
-40
Myc
-55
α-Tubulin
-55
S7m
Mr (K)
HOIP
-130
ILK
-55
α-Tubulin
-55
S8a
Mr (K)
His
-75
GST
-170
-130
-100
70
55
-25
WCL
His
-70
S8b
Mr (K)
SMURF1
-100
Myc
-85
-70
-50
-30
-20
WCL
SMURF1
-100
S8c
Mr (K)
Myc
-130
Flag
-100
-70
-55
-40
Myc (IP)
-130
Flag (IP)
-100
-70
-55
-40
S8h
WCL
IP Myc
Mr (K)
Flag
HOIP
-130
Myc
Smurf1
-100
-70
Flag
Smurf1
-100
-70
S8j
Mr (K)
SMAD1
-55
Flag (IP)
-100
Flag
-100
SMAD1
-55
Myc
-130
HA
-55
S8e
Mr (K)
M1 Ub
-170
-130
-100
-70
Flag (IP)
-100
-70
Myc
HOIP
-130
-100
HA
HOIL1
-55
Flag
SMURF1
-100
-70
Myc
OTULIN
-40
S8g
Mr (K)
M1 Ub
-170
-130
-100
-70
-55
-40
Myc
-100
-70
-55
-40
Flag
-170
-130
-100
-70
HA
-55
-40
S8i
Mr (K)
GFP
- 25
Flag (IP)
- 70
GFP
- 35
- 25
Flag
- 100
- 70
Myc
- 130
HA
- 55
S9d
Mr (K)
HOIP
- 130
HOIL1
- 55
SHARPIN
- 40
β-actin
- 40
S9f
Mr (K)
M1 Ub
-170
-130
-100
-70
Flag (IP)
-100
-70
M1 Ub
-170
-130
-100
-70
Flag
-100
-70
Myc
-130
-100
HA
-55
S9k
Mr (K)
SMAD1
- 70
- 55
SMURF1
- 100
- 70
β-actin
- 40
S9l
Mr (K)
HOIP
- 130
SMURF1
- 100
MEKK2
- 100
- 70
SMAD1/5
- 55
β-actin
- 40
