# Supplementary material for: Systematic HOIP interactome profiling reveals critical roles of linear ubiquitination in tissue homeostasis
Source: Nat Commun. 2024 Apr 6;15:2974. doi: 10.1038/s41467-024-47289-2 (PMC10998861; doi:10.1038/s41467-024-47289-2)
Supplement: Supplementary file 3 — Description of Additional Supplementary Files [file 41467_2024_47289_MOESM3_ESM.pdf]

Description of Additional Supplementary Files

Supplementary Data 1: iBAQ of biological replicates across tissues and domains, related to Supplementary Fig. 1.

Supplementary Data 2: HOIP interactions identified by differential analysis, related to Fig. 1.

Supplementary Data 3: HOIP interaction partners were enriched in biological process terms, related to Supplementary Fig. 2.

a) Total biological process list for HOIP interactors across tissues.

b) Manual selected biological process list related to Supplementary Fig. 2a.

Supplementary Data 4: Tissue-shared and tissue-specific HOIP PPIs, related to Fig. 2.

a) Statistical result for tissue-specific and tissue-shared interactors across nine tissues. b) Total HOIP-interacting proteins list for tissue-specific and tissue-shared interactors across nine tissues.

c) Tissue-shared HOIP interaction partners were enriched in biological process terms refer to Fig. 2c.

d) Intra tissue-shared HOIP interaction partners curated from STRING database refer to Fig. 2c.

e) Tissue-specific HOIP interaction partners were enriched in biological process terms refer to Fig. 2e.

f) Intra tissue-specific HOIP interaction partners curated from STRING database refer to Fig. 2e.

g) Enriched proteins in genetic diseases across tissues.

Supplementary Data 5: HOIP domains mediated PPIs

a) Biological process list for all HOIP interaction partners identification of different domains, related to Supplementary Fig. 3a.

b) Manual selected representative biological processes, related to Fig. 3a.

c) Manual selected biological process list of domain-specific HOIP PPIs, related to Supplementary Fig. 4a.

d) GO terms specifically enriched in manual selected tissues and domains.

Supplementary Data 6: HOIP PPIs in lungs from LPS-induced sepsis models, related to Supplementary Fig. 5.

a) Total HOIP-interacting proteins list in lungs from LPS-induced sepsis models.

b) Manual selected biological process list of LPS-specific HOIP PPIs, related to Supplementary Fig. 5d.

c) Total HOIP-PUB interacting proteins list in lungs from LPS-induced sepsis models, related to Supplementary Fig. 5d.

d) Total HOIP-NZF interacting proteins list in lungs from LPS-induced sepsis models, related to Supplementary Fig. 5d.

e) Total HOIP-UBA interacting proteins list in lungs from LPS-induced sepsis models, related to Supplementary Fig. 5d.

f) Total HOIP-RBR interacting proteins list in lungs from LPS-induced sepsis models, related to Supplementary Fig. 5d.

Supplementary Data 7: HOIP PPIs involved in the Kinase, TF, E3 and DUBs, related to Supplementary Fig. 3.

a) Statistical result of HOIP PPIs involved in indicated protein classes, related to Supplementary Fig. 6a

b) Gene list of HOIP PPIs involved in indicated protein classes

c) The list of kinases interacting with HOIP across tissues and domains, related to Supplementary Fig. 6c

Supplementary Data 8: oligonucleotides.
