# Supplementary material for: Systematic HOIP interactome profiling reveals critical roles of linear ubiquitination in tissue homeostasis
Source: Nat Commun. 2024 Apr 6;15:2974. doi: 10.1038/s41467-024-47289-2 (PMC10998861; doi:10.1038/s41467-024-47289-2)
Supplement: Supplementary file 12 — Reporting Summary [file 41467_2024_47289_MOESM12_ESM.pdf]

nature portfolio

Corresponding author(s): Lingqiang Zhang & Cui Hua Liu

Last updated by author(s): Mar 22, 2024

# Reporting Summary

Nature Portfolio wishes to improve the reproducibility of the work that we publish. This form provides structure for consistency and transparency in reporting. For further information on Nature Portfolio policies, see our Editorial Policies and the Editorial Policy Checklist.

## Statistics

For all statistical analyses, confirm that the following items are present in the figure legend, table legend, main text, or Methods section.

| n/a | Confirmed | |
|---|---|---|
| ☐ | ☒ | The exact sample size (*n*) for each experimental group/condition, given as a discrete number and unit of measurement |
| ☐ | ☒ | A statement on whether measurements were taken from distinct samples or whether the same sample was measured repeatedly |
| ☐ | ☒ | The statistical test(s) used AND whether they are one- or two-sided<br>*Only common tests should be described solely by name; describe more complex techniques in the Methods section.* |
| ☒ | ☐ | A description of all covariates tested |
| ☐ | ☒ | A description of any assumptions or corrections, such as tests of normality and adjustment for multiple comparisons |
| ☐ | ☒ | A full description of the statistical parameters including central tendency (e.g. means) or other basic estimates (e.g. regression coefficient) AND variation (e.g. standard deviation) or associated estimates of uncertainty (e.g. confidence intervals) |
| ☒ | ☐ | For null hypothesis testing, the test statistic (e.g. *F*, *t*, *r*) with confidence intervals, effect sizes, degrees of freedom and *P* value noted<br>*Give P values as exact values whenever suitable.* |
| ☒ | ☐ | For Bayesian analysis, information on the choice of priors and Markov chain Monte Carlo settings |
| ☒ | ☐ | For hierarchical and complex designs, identification of the appropriate level for tests and full reporting of outcomes |
| ☒ | ☐ | Estimates of effect sizes (e.g. Cohen's *d*, Pearson's *r*), indicating how they were calculated |

*Our web collection on statistics for biologists contains articles on many of the points above.*

## Software and code

Policy information about availability of computer code

| | |
|---|---|
| Data collection | Quantitative PCR: QuantStudio 3 (Thermo Fisher Scientific).<br>Immunohistochemistry:  Freezing microtome (RWD Minux FS800, China).<br>Immunofluorescence: Fluorescence microscopy (NikonA1R), Pannoramic MIDI scanner (3 DHISTECH, Hungary).<br>Micro CT analysis: Inveon MM CT (SIEMENS, Munich, Germany).<br>Biomechanical test: UniVert (CellScale Biomaterials Testing, Canada).<br>Mass spectrometry: LC-MS/MS system that consisted of an online Easy-nLC 1200 nano-HPLC system (Thermo Fisher Scientific) and the Orbitrap Fusion lumos mass spectrometer (Thermo Fisher Scientific). |
| Data analysis | Image analysis: ImageJ (v1.8.0), CaseViewer (v2.4.0.119028), NIS-Elements AR (4.40.00), Bioquant Osteo software (v20.5.6).<br>Micro CT analysis: Inveon Research Workplace (v1.0), COBRA_Exxim (v1.0).<br>Statistical analysis: GraphPad Prism (v8.0.2).<br>Mass spectrometry analysis: maxquant (2.0.3.0), ProstaR and DAPAR (v1.30.7), ClusterProfiler (v4.6.2), Cytoscape (v3.9.1). |

For manuscripts utilizing custom algorithms or software that are central to the research but not yet described in published literature, software must be made available to editors and reviewers. We strongly encourage code deposition in a community repository (e.g. GitHub). See the Nature Portfolio guidelines for submitting code & software for further information.

## Data

Policy information about availability of data

All manuscripts must include a data availability statement. This statement should provide the following information, where applicable:

- Accession codes, unique identifiers, or web links for publicly available datasets
- A description of any restrictions on data availability
- For clinical datasets or third party data, please ensure that the statement adheres to our policy

Raw mass spectrometry data generated in this study have been deposited in ProteomeXchange Consortium via the iProX repository with the identifier PXD043322 (https://proteomecentral.proteomexchange.org/cgi/GetDataset?ID=PXD043322) and these data have been publicly released. All the data supporting the findings of this study are available within the article and its Supplementary Information. Source data have been provided within this paper and deposited in the "figshare" (https://figshare.com/) and the DOIs for "figshare" are provided (10.6084/m9.figshare.25303996, 10.6084/m9.figshare.25040426).

## Research involving human participants, their data, or biological material

Policy information about studies with human participants or human data. See also policy information about sex, gender (identity/presentation), and sexual orientation and race, ethnicity and racism.

| | |
|---|---|
| Reporting on sex and gender | None |
| Reporting on race, ethnicity, or other socially relevant groupings | None |
| Population characteristics | None |
| Recruitment | None |
| Ethics oversight | None |

Note that full information on the approval of the study protocol must also be provided in the manuscript.

# Field-specific reporting

Please select the one below that is the best fit for your research. If you are not sure, read the appropriate sections before making your selection.

☒ Life sciences ☐ Behavioural & social sciences ☐ Ecological, evolutionary & environmental sciences

For a reference copy of the document with all sections, see nature.com/documents/nr-reporting-summary-flat.pdf

# Life sciences study design

All studies must disclose on these points even when the disclosure is negative.

| | |
|---|---|
| Sample size | All sample sizes are indicated in the figure legends. No sample size calculation was performed. To determine sample sizes in vitro, the number of samples that were available and the number of samples required to establish statistical significance were taken into consideration. For in vivo, the sample size was determined based on our previous work (e.g. Fu et al., Mol cell, 2021). For mass spectrometry, the sample size was determined based on previous proteomics research (e.g. Li et al., Nat Commun, 2018; Wang et al., Nat Commun, 2018). |
| Data exclusions | No data is excluded if the experiments were successfully performed. |
| Replication | We have indicated the number of independent experiments performed in the Methods section or the figure legends. |
| Randomization | Samples or animals were allocated randomly into experimental groups. |
| Blinding | For in vitro study, investigators were not blinded to the sample identities during data collection since the readouts were quantitative and not prone to subjective judgment of investigators. For in vivo study, mice experiments and statistical analysis were performed by independent researchers in a blinded manner. |

# Reporting for specific materials, systems and methods

We require information from authors about some types of materials, experimental systems and methods used in many studies. Here, indicate whether each material, system or method listed is relevant to your study. If you are not sure if a list item applies to your research, read the appropriate section before selecting a response.

## Materials & experimental systems

| n/a | Involved in the study |
| --- | --- |
| ☐ | ☒ Antibodies |
| ☐ | ☒ Eukaryotic cell lines |
| ☒ | ☐ Palaeontology and archaeology |
| ☐ | ☒ Animals and other organisms |
| ☒ | ☐ Clinical data |
| ☒ | ☐ Dual use research of concern |
| ☒ | ☐ Plants |

## Methods

| n/a | Involved in the study |
| --- | --- |
| ☒ | ☐ ChIP-seq |
| ☒ | ☐ Flow cytometry |
| ☒ | ☐ MRI-based neuroimaging |

# Antibodies

Antibodies used

All of the antibodies were used according to the manufacturer's instructions and based on previous experience in the laboratory. The following commercial antibodies have been used: GAPDH (Santa Cruz, Cat# sc-365062, RRID:AB_10847862, 1:2000 for IB), β-actin (Proteintech, Cat# 66009-1-Ig, RRID:AB_2687938, 1:4000 for IB), Myc (MBL, Cat# M047-3, RRID:AB_591112, 1:4000 for IB, 1:500 for IP), Flag (MBL, Cat# M185-3, RRID:AB_10950447, 1:1000 for IB, 1:500 for IP), HA (MBL, Cat# M180-3, RRID:AB_10951811, 1:1000 for IB), HOIP (Abcam, Cat# ab46322, RRID:AB_945269, 1:300 for IB), HOIP (Abcam, Cat# ab125189, RRID: AB_10976137, 1:500 for IB, 1:200 for IP), HOIL-1 (Sigma-Aldrich Cat# HPA024185, RRID:AB_1845673, 1:300 for IB), SHARPIN (Proteintech, Cat# 14626-1-AP, RRID:AB_2187734, 1:1000 for IB), OTULIN ( Cell Signaling, Cat# 14127, RRID:AB_2576213, 1:1000 for IB), SMURF1(Abcam, Cat# ab57573, RRID:AB_945548 , 1:500 for IB), SMURF1 (Abnova, Cat#H00057154-M01, RRID:AB_566195, 1:500 for IB, 1:200 for IP), SMAD1/5 (Abcam, Cat# ab75273, RRID:AB_1310686, 1:500 for IB), SMAD1(Santa Cruz Biotechnology, Cat# sc-7965, RRID:AB_628261, 1:400 for ICC), MEKK2 (Proteintech, Cat# 55106-1-AP, RRID:AB_11064604, 1:1000 for IB), AP2B1 (Proteintech, Cat# 15690-1-AP, RRID:AB_2056351, 1:1000 for IB), AP2M1 (Proteintech, Cat# 27355-1-AP, RRID:AB_2880853, 1:1000 for IB), RUVBL1 (Proteintech, Cat# 10210-2-AP, RRID:AB_2184405, 1:1500 for IB), RUVBL2 (Proteintech, Cat# 10195-1-AP, RRID:AB_2184679, 1:1500 for IB), SYN1 (Proteintech, Cat# 20258-1-AP, RRID:AB_2800493, 1:1500 for IB), HAP1 (Proteintech, Cat# 25133-1-AP, RRID:AB_2879915, , 1:500 for IB), MYL3 (Proteintech, Cat# 10913-1-AP, RRID:AB_2147607, 1:800 for IB), PRKAA2 (Proteintech, Cat# 18167-1-AP, RRID:AB_10695046, 1:1000 for IB), TRAF1(Proteintech, Cat# 26845-1-AP, RRID:AB_2880655, 1:1500 for IB), TRAF2 (Proteintech, Cat# 26846-1-AP, RRID:AB_2880656, 1:1500 for IB), CDHR5 (Proteintech, Cat# 25619-1-AP, RRID:AB_2880164, 1:1000 for IB), CSRP3 (Proteintech, Cat# 10721-1-AP, RRID:AB_2292475, 1:1000 for IB), ABCB1 (Proteintech, Cat# 22336-1-AP, RRID:AB_2833023, 1:2000 for IB), SCP2 (Proteintech, Cat# 23006-1-AP, RRID: AB_2879197, 1:1000 for IB), STAT1 (Proteintech, Cat# 10144-2-AP, RRID:AB_2286875, 1:3000 for IB, 1:500 for IP), β-Catenin (Abcam, Cat# ab32572, RRID:AB_725966, 1:1000 for IB, 1:50 for IP), FXR1 (Proteintech, Cat#13194-1-AP, RRID:AB_2110702, 1:1000 for IB), Ubiquitin (Cell Signaling Technology Cat# 20326, RRID:AB_3064918, 1:1000 for IB), M1 Ub (Lifesensors, Cat# AB130, RRID:AB_2576211, 1:300 for IB, 1:100 for IHC), GFP (Proteintech, Cat# 50430-2-AP, RRID:AB_11042881, 1:1000 for IB, 1:1000 for IF), ILK (Proteintech, Cat# 12955-1-AP, RRID:AB_2127053, 1:200 for IB), ILK (Proteintech, Cat# 67724-1-Ig, RRID:AB_2882910, 1:500 for IB, 1:50 for IP), ILK ((Santa Cruz Biotechnology, Cat# sc-137221, RRID:AB_2127074, 1:500 for IB, 1:50 for IF), αTubulin (Biodragon, Cat# B1052, RRID: AB_2936302, 1:1000 for IB), FAK (Abclonal, Cat#A11131, RRID:AB_2758423, 1:1000 for IB), FAK (Y397) (Abclonal, Cat# AP0302, RRID:AB_2771470, 1:500 for IB), Vinculin (Proteintech, Cat# 26520-1-AP, RRID:AB_2868558, 1:300 for IF), α-parvin (Proteintech, Cat# 55268-1-AP; RRID:AB_10951112, 1:500 for IB); Goat anti-Rabbit IgG (H+L) Secondary Antibody (Thermo Fisher Scientific, Cat# 65-6120, RRID:AB_2533967, 1:2500 for IB, 1:1000 for IHC); Goat anti-Mouse IgG (H+L) Secondary Antibody, HRP (Thermo Fisher Scientific, Cat# 31430, RRID:AB_228307, 1:2500 for IB, 1:1000 for IHC); Alexa Fluor 488 Recombinant Polyclonal Antibody (Thermo Fisher Scientific, Cat# 710369, RRID:AB_2532697, 1:1000 for IF); Goat anti-Rabbit IgG (H+L) Cross-Adsorbed Secondary Antibody, Alexa Fluor™ 594 (Thermo Fisher Scientific, Cat# A-11012, RRID:AB_2534079, 1:1000 for IF); Donkey anti-Rabbit IgG (H+L) Highly Cross-Adsorbed Secondary Antibody, Alexa Fluor™ 647 (Thermo Fisher Scientific, Cat# A-31573, RRID:AB_2536183 1:1000 for IF).

Validation

All the antibodies used in this study have been validated as reported in manufacture's website:
Anti-GAPDH antibody, Cat# sc-365062, https://www.scbt.com/p/gapdh-antibody-g-9?requestFrom=search;
Anti-β-actin antibody, Cat# 66009-1-Ig, https://www.ptgcn.com/products/Pan-Actin-Antibody-66009-1-Ig.htm;
Anti-Myc antibody, Cat# M047-3, https://products.mblintl.com/products/m047-3/;
Anti-Flag antibody, Cat# M185-3, https://www.mblbio.com/bio/g/dtl/A/index.html?pcd=M185-3L;
Anti-HA antibody, Cat# M180-3, https://www.mblbio.com/bio/g/dtl/A/?pcd=M180-3;
Anti-HOIP antibody, Cat# ab46322, https://www.abcam.com/products/primary-antibodies/rnf31hoip-antibody-ab46322.html;
Anti-HOIP antibody, Cat# ab125189, https://www.abcam.com/products/primary-antibodies/rnf31hoip-antibody-ab125189.html;
Anti-HOIL-1 antibody, Cat# HPA024185, https://www.sigmaaldrich.cn/CN/zh/product/sigma/hpa024185;
Anti-SHARPIN antibody, Cat# 14626-1-AP, https://www.ptgcn.com/products/SHARPIN-Antibody-14626-1-AP.htm;
Anti-OTULIN antibody, Cat# 14127, https://www.cellsignal.com/products/primary-antibodies/otulin-antibody/14127;
Anti-SMURF1 antibody, Cat# ab57573, https://www.abcam.com/products/primary-antibodies/smurf1-antibody-1d7-ab57573.html;
Anti-SMURF1 antibody, Cat#H00057154-M01, https://www.novusbio.com/products/smurf1-antibody-1d7_h00057154-m01;
Anti-SMAD1/5 antibody, Cat# ab75273, https://www.abcam.com/products/primary-antibodies/smad1smad5-antibody-af10b7-ab75273.html;
Anti-SMAD1 antibody, Cat# sc-7965, https://www.scbt.com/p/smad1-antibody-a-4?requestFrom=search;
Anti-MEKK2 antibody, Cat# 55106-1-AP, https://www.ptgcn.com/products/MAP3K2-Antibody-55106-1-AP.htm;
Anti-AP2B1 antibody, Cat# 15690-1-AP, https://www.ptgcn.com/products/AP2B1-Antibody-15690-1-AP.htm;
Anti-AP2M1 antibody, Cat# 27355-1-AP, https://www.ptgcn.com/products/AP50-Antibody-27355-1-AP.htm;
Anti-RUVBL1 antibody, Cat# 10210-2-AP, https://www.ptgcn.com/products/RUVBL1-Antibody-10210-2-AP.htm;
Anti-RUVBL2 antibody, Cat# 10195-1-AP, https://www.ptgcn.com/products/RUVBL2-Antibody-10195-1-AP.htm;
Anti-SYN1 antibody, Cat# 20258-1-AP, https://www.ptgcn.com/products/SYN1-Specific-Antibody-20258-1-AP.htm;
Anti-HAP1 antibody, Cat# 25133-1-AP, https://www.ptgcn.com/products/HAP1-Antibody-25133-1-AP.htm;
Anti-MYL3 antibody, Cat# 10913-1-AP, https://www.ptgcn.com/products/MYL3-Antibody-10913-1-AP.htm;
Anti-PRKAA2 antibody, Cat# 18167-1-AP, https://www.ptgcn.com/products/PRKAA2-Antibody-18167-1-AP.htm;

Anti-TRAF1antibody, Cat# 26845-1-AP, https://www.ptgcn.com/products/TRAF1-Antibody-26845-1-AP.htm;
Anti-TRAF2 antibody, Cat# 26846-1-AP, https://www.ptgcn.com/products/TRAF2-Antibody-26846-1-AP.htm;
Anti- Anti-CDHR5 antibody, Cat# 25619-1-AP, https://www.ptgcn.com/products/MUPCDH-Antibody-25619-1-AP.htm;
Anti-CSRP3 antibody, Cat# 10721-1-AP, https://www.ptgcn.com/products/CSRP3-Antibody-10721-1-AP.htm;
Anti-ABCB1 antibody, Cat# 22336-1-AP, https://www.ptgcn.com/products/ABCB1-Antibody-22336-1-AP.htm;
Anti-SCP2 antibody, Cat# 23006-1-AP, https://www.ptgcn.com/products/SCP2-Antibody-23006-1-AP.htm;
Anti-STAT1 antibody, Cat# 10144-2-AP, https://www.ptgcn.com/products/STAT1-Antibody-10144-2-AP.htm;
Anti-β-Catenin, Cat# ab32572, https://www.abcam.com/products/primary-antibodies/beta-catenin-antibody-e247-chip-grade-ab32572.html;
Anti-FXR1 antibody, Cat#13194-1-AP, https://www.ptgcn.com/products/FXR1-Antibody-13194-1-AP.htm;
Anti-Ubiquitin antibody, Cat# 20326, https://www.cellsignal.com/products/primary-antibodies/ubiquitin-e6k4y-xp-rabbit-mab/20326;
Anti-M1 Ub, Cat# AB130, https://lifesensors.com/product/ab130-linear-polyubiquitin-antibody-mab-clone-lub9/;
Anti-GFP antibody, Cat# 50430-2-AP, https://www.ptgcn.com/products/eGFP-Antibody-50430-2-AP.htm;
Anti-ILK antibody, Cat# 12955-1-AP, https://www.ptgcn.com/products/ILK-Antibody-12955-1-AP.htm;
Anti-ILK antibody, Cat# 67724-1-Ig, https://www.ptgcn.com/products/ILK-Antibody-67724-1-Ig.htm;
Anti-ILK, Cat# sc-137221, https://www.scbt.com/p/ilk-antibody-e-2?requestFrom=search;
Anti-αTubulin, Cat# B1052, https://www.biodragon.cn/cn/goods/goodsView?GoodsId=12552;
Anti-FAK, Cat#A11131, https://abclonal.com/catalog-antibodies/FAKRabbitmAb/A11131;
Anti-FAK (Y397), Cat# AP0302, https://abclonal.com/catalog-antibodies/PhosphoFAKY397RabbitpAb/AP0302;
Anti-Vinculin antibody, Cat# 26520-1-AP, https://www.ptgcn.com/products/Vinculin-Antibody-26520-1-AP.htm;
Anti-α-parvin antibody, Cat# 55268-1-AP, https://www.ptgcn.com/products/PARVA-Antibody-55268-1-AP.htm;
Goat anti-Rabbit IgG (H+L) Secondary Antibody, Cat# 65-6120, https://www.thermofisher.cn/cn/zh/antibody/product/Goat-anti-Rabbit-IgG-H-L-Secondary-Antibody-Polyclonal/65-6120;
Goat anti-Mouse IgG (H+L) Secondary Antibody, HRP, Cat# 31430, https://www.thermofisher.cn/cn/zh/antibody/product/Goat-anti-Mouse-IgG-H-L-Secondary-Antibody-Polyclonal/31430;
Alexa Fluor 488 Recombinant Polyclonal Antibody, Cat# 710369, https://www.thermofisher.cn/cn/zh/antibody/product/Alexa-Fluor-488-Antibody-Recombinant-Polyclonal/710369;
Goat anti-Rabbit IgG (H+L) Cross-Adsorbed Secondary Antibody, Alexa Fluor™ 594, Cat# A-11012, https://www.thermofisher.cn/cn/zh/antibody/product/Goat-anti-Rabbit-IgG-H-L-Cross-Adsorbed-Secondary-Antibody-Polyclonal/A-11012;
Donkey anti-Rabbit IgG (H+L) Highly Cross-Adsorbed Secondary Antibody, Alexa Fluor™ 647, Cat# A-31573, https://www.thermofisher.cn/cn/zh/antibody/product/Donkey-anti-Rabbit-IgG-H-L-Highly-Cross-Adsorbed-Secondary-Antibody-Polyclonal/A-31573.

## Eukaryotic cell lines

Policy information about cell lines and Sex and Gender in Research

**Cell line source(s)**

HEK293T cells, ATCC Cat# CRL-11268,
Hela cells, ATCC, Cat# CCL-2,
MC3T3-E1 cells, ATCC Cat# CRL-2593.
BMSC were isolated from femurs of 8-week-old male mice.

**Authentication**

Commercialized cells are authenticated by STR profiling.

**Mycoplasma contamination**

All cells tested negative for mycoplasma contamination.

**Commonly misidentified lines** (See ICLAC register)

No cell lines adopted in this study is listed in the database of commonly misidentified cell lines maintained by ICLAC.

## Animals and other research organisms

Policy information about studies involving animals; ARRIVE guidelines recommended for reporting animal research, and Sex and Gender in Research

**Laboratory animals**

All mice were on a C57BL/6 background and maintained under specific pathogen-free conditions in individually ventilated cages (12 h light/dark cycle, 50% relative humidity, between 25 and 27 °C). 8-week-old HoiploxP/loxP mice were obtained from RIKEN (cat#RBRC09483). 8-week-old wild-type mice and transgenic mice expressing Cre recombinase under control of the Osx (also known as Sp7) promoter (C57BL/6-Sp7tm1(icre)/Bcgen) were purchased from BIOCYTOGEN (cat#110131). HoiploxP/loxP mice (approximately 8–12 weeks) and Osx-Cre mice were used to generate Hoip osteoblast-specific deletion mice (HoiploxP/loxP Osx-Cre +).

**Wild animals**

no wild animals were used in the study.

**Reporting on sex**

Female and male mice were both used in this study. For bone phenotype analysis, we mainly analyzed male mice, since many studies have shown that the estrogen impacts bone formation (Almeida et al., Physiol Rev, 2017).

**Field-collected samples**

no field collected samples were used in the study.

**Ethics oversight**

The Institutional Animal Care and Use Committee of the Beijing Institute of Lifeomics is responsible for ethical compliance approval of all animal protocols (IACUC-DWZX-2023-P503).

Note that full information on the approval of the study protocol must also be provided in the manuscript.

## Plants

| | |
|---|---|
| Seed stocks | None |
| Novel plant genotypes | None |
| Authentication | None |
